# Commentary: CD22 blockade restores homeostatic microglial phagocytosis in ageing brains

**DOI:** 10.3389/fimmu.2019.01301

**Published:** 2019-06-06

**Authors:** Penghui Wei, Jianjun Li

**Affiliations:** Department of Anesthesiology, Qilu Hospital of Shandong University (Qingdao), Qingdao, China

**Keywords:** CD22 blockade, microglial phagocytosis, immunotherapy, neurodegenerative diseases, POCD

Recently, a ground-breaking study on microglia being a potential target for immunotherapy in neurodegenerative diseases was reported in Nature (1). Pluvinage et al. combined CRISPR–Cas9 knockout screens with RNA sequencing analysis to identify approximately 3,000 genes. They finally discovered that CD22, a canonical receptor typically expressed on B-cells (2), was significantly upregulated in microglia of the central nervous system (CNS) of an aged mouse, and approximately three times more in aged microglia than young microglia. Interestingly, the authors showed that CD22 was a negative regulator of microglial phagocytosis, and blocking CD22 in CNS either by specific antibody or by genetic ablation promoted the clearance of extracellular oligomeric amyloid-β [Aβ, neuropathological hallmark of Alzheimer's disease (AD)] and α-synuclein fibrils (pathological hallmark of Parkinson's disease) *in vivo*, and improved hippocampal-dependent learning and memory performance. Furthermore, they found that the mechanism underlying microglial CD22-blocking-mediated neuroprotection may involve an increase in the number of hippocampal dentate granule neurons expressing c-FOS and phospho-cAMP response element-binding protein. The research represents a breakthrough in the field of treatment for neurodegenerative diseases. The immunotherapy conducted by microglial CD22 may be a potential therapeutic strategy to restore and improve age-related cognitive impairment.

Perioperative neurocognitive disorder, an age-related neurodegenerative disease, is a highly prevalent condition with significant effects on the prognosis of elderly individuals undergoing anesthesia and surgery, including acute post-operative delirium and long-lasting post-operative cognitive dysfunction (POCD) (3, 4). The incidence of POCD was reportedly between 25 and 40% among elderly patients at the point of discharge, and it could lead to a significantly higher mortality rate (5). The exact mechanism of POCD remains elusive and there is no agreement on the efficiency of current treatments such as anti-inflammatory, antioxidant and neuroprotective agents (6). Aβ and phosphorylation of tau protein, secondary to anesthesia and surgery, are believed to be pathological hallmarks of POCD (7). Normal microglia engulf and degrade Aβ and tau protein by phagocytosis; however, the change in engulfment of microglia is associated with age and the aged microglia become more proinflammatory and less phagocytic (1, 8). Therefore, we speculated that the age-related deterioration of microglial phagocytosis could be the potential mechanism underlying POCD. Immunotherapy mediated by the restoration of microglial phagocytosis could provide a new path in preventing and treating POCD.

Immunotherapy induced by selective blockade of some immune checkpoints, such as PD-1/PD-L1, is commonly utilized to mobilize the immune system against a variety of malignancies (9). Research examining the use of immunotherapy in neurodegenerative diseases, such as AD and POCD, seems promising but appears relatively under-developed as a field. A ground-breaking report in Nature Medicine in 2015 showed that the PD-1 pathway blockade evoked an interferon-γ-associated systemic immune response, induced CNS recruitment of myeloid cell, and restrained accumulation of cerebral Aβ, which contributed to improvement of cognitive performance in heterozygous 5XFAD transgenic mouse models of AD (10). A recent study has also demonstrated that immune checkpoint blockade targeting the PD-1/PD-L1 pathway resulted in increased immunomodulatory monocyte-derived macrophages within the brain parenchyma and combated cognitive impairment in mouse models of both AD and tauopathy (11). However, the available evidence is inconsistent. Gomez-Nicol et al. observed no apparent impact of PD-1 deficiency on murine prion disease (ME7 strain). On the contrary, they found a slight exacerbation of the cognitive performance of ME7 mice upon PD-1 deficiency (12). The conflicting findings may attribute to different animal models presenting neurodegenerative diseases. Another potential reason could be non-specific expression of PD-1 on immune cells in the aged mouse, which is widely expressed in dendritic cells, B-cells, activated T-cells (both CD4+ and CD8+), and natural killer cells (9). The CD22 blockade appears to be more promising than PD-1/PD-L1 blockade, and may exhibit better efficiency and lesser side effects as a therapeutic strategy in neurodegenerative diseases. Although CD22 is typically expressed in B-cells, it is specifically upregulated in aged microglia (1). Pluvinage et al. observed that blocking CD22 specifically in the CNS via cerebral injection, rather than blocking CD22 systemically via intraperitoneal injection, contributed to cognitive improvement in aged mice that showed that peripheral B-cells did not mediate neuroprotective effect of anti-CD22 treatment (1). Moreover, pro-phagocytic effect of anti-CD22 treatment was mainly mediated by resident microglia and not the infiltrating peripheral macrophages (13). Furthermore, the upregulated CD22 expression in microglia depends on age. The study showed that aged microglia increased the number of CD22 surface molecules approximately three times more than young microglia, and CD22 blockade treatment had effects that were more significant in the CNS of the aged mouse than that of the younger mouse (1). Above all, aged microglia specifically expressing CD22 in CNS could be a potential and viable molecular target in neurodegenerative diseases, especially for AD and POCD. However, the effect of anti-CD22 treatment on age-related diseases needs to further studies involving different animal models.

## Author Contributions

PW drafted the manuscript. JL revised the manuscript. All authors read and approved the final manuscript.

### Conflict of Interest Statement

The authors declare that the research was conducted in the absence of any commercial or financial relationships that could be construed as a potential conflict of interest.

## References

[1] Pluvinage JV Haney MS Smith BAH Sun J Iram T Bonanno L. CD22 blockade restores homeostatic microglial phagocytosis in ageing brains. Nature. (2019) 568:187–92. 10.1038/s41586-019-1088-4 30944478 PMC6574119

[2] Macauley MS Crocker PR Paulson JC. Siglec-mediated regulation of immune cell function in disease. Nat Rev Immunol. (2014) 14:653–66. 10.1038/nri3737 25234143 PMC4191907

[3] Evered L Silbert B Knopman DS Scott DA DeKosky ST Rasmussen LS. Recommendations for the nomenclature of cognitive change associated with anaesthesia and surgery-2018. Anesthesiology. (2018) 129:872–9. 10.1097/ALN.0000000000002334 30325806

[4] Xiang X Yu Y Tang X Chen M Zheng Y Zhu S. Transcriptome profile in hippocampus during acute inflammatory response to surgery: toward early stage of PND. Front Immunol. (2019) 10:149. 10.3389/fimmu.2019.00149 30804943 PMC6370675

[5] Skvarc DR Berk M Byrne LK Dean OM Dodd S Lewis M. Post-operative cognitive dysfunction: an exploration of the inflammatory hypothesis and novel therapies. Neurosci Biobehav Rev. (2018) 84:116–33. 10.1016/j.neubiorev.2017.11.011 29180259

[6] Wei P Yang F Zheng Q Tang W Li J. The potential role of the NLRP3 inflammasome activation as a link between mitochondria ROS generation and neuroinflammation in postoperative cognitive dysfunction. Front Cell Neurosci. (2019) 13:73. 10.3389/fncel.2019.00073 30873011 PMC6401615

[7] Xie Z McAuliffe S Swain CA Ward SA Crosby CA Zheng H. Cerebrospinal fluid abeta to tau ratio and postoperative cognitive change. Ann Surg. (2013) 258:364–9. 10.1097/SLA.0b013e318298b077 23732272 PMC3880113

[8] Marsh SE Abud EM Lakatos A Karimzadeh A Yeung ST Davtyan H. The adaptive immune system restrains Alzheimer's disease pathogenesis by modulating microglial function. Proc Natl Acad Sci USA. (2016) 113:E1316–25. 10.1073/pnas.1525466113 26884167 PMC4780638

[9] Constantinidou A Alifieris C Trafalis DT. Targeting programmed cell death−1 (PD-1) and ligand (PD-L1): a new era in cancer active immunotherapy. Pharmacol Ther. (2019) 194:84–106. 10.1016/j.pharmthera.2018.09.008 30268773

[10] Baruch K Deczkowska A Rosenzweig N Tsitsou-Kampeli A Sharif AM Matcovitch-Natan O. PD-1 immune checkpoint blockade reduces pathology and improves memory in mouse models of Alzheimer's disease. Nat Med. (2016) 22:135–7. 10.1038/nm.4022 26779813

[11] Rosenzweig N Dvir-Szternfeld R Tsitsou-Kampeli A Keren-Shaul H Ben-Yehuda H Weill-Raynal P. PD-1/PD-L1 checkpoint blockade harnesses monocyte-derived macrophages to combat cognitive impairment in a tauopathy mouse model. Nat Commun. (2019) 10:465. 10.1038/s41467-019-08352-5 30692527 PMC6349941

[12] Obst J Mancuso R Simon E Gomez-Nicola D. PD-1 deficiency is not sufficient to induce myeloid mobilization to the brain or alter the inflammatory profile during chronic neurodegeneration. Brain Behav Immun. (2018) 73:708–16. 10.1016/j.bbi.2018.08.006 30086399 PMC6191933

[13] Bennett ML Bennett FC Liddelow SA Ajami B Zamanian JL Fernhoff NB. New tools for studying microglia in the mouse and human CNS. Proc Natl Acad Sci USA. (2016) 113:E1738–46. 10.1073/pnas.1525528113 26884166 PMC4812770

